# Complete genome sequence of *Francisella salimarina* GTC 22824 isolated from coastal seawater in Osaka, Japan

**DOI:** 10.1128/mra.00286-26

**Published:** 2026-06-15

**Authors:** Masahiro Hayashi, Yushi Naka, Hiromi Machida, Masanori Miyata, Jun Yonetamari, Yoshinori Muto, Motoki Takagi, Kaori Tanaka

**Affiliations:** 1 Institute for Glyco-core Research iGCORE, Gifu University 12785, Gifu City, Gifu, Japan; 2 Division of Anaerobe Research, Life Science Research Center, Gifu University 201111, Gifu City, Gifu, Japan; 3 Gifu University Center for Conservation of Microbial Genetic Resource, Gifu, Japan; 4 MIZUKEN Co. Ltd., Sakai City, Osaka, Japan; 5 Division of Clinical Laboratory, Gifu University Hospital 476117, Gifu, Japan; 6 United Graduate School of Drug Discovery and Medical Information Sciences, Gifu University 12785, Gifu City, Gifu, Japan; 7 JeiserBio inc., Yokohama City, Kanagawa, Japan; Portland State University, Portland, Oregon, USA

**Keywords:** *Francisella salimarina*, genomr

## Abstract

Herein, we report the complete genome sequence of *Francisella salimarina* GTC 22824, isolated from coastal seawater in Osaka, Japan. The genome comprises a circular chromosome measuring 1,991,123 bp.

## ANNOUNCEMENT

*Francisella salimarina* has been isolated from environmental and clinical sources (1, 2). However, the complete genome sequence of *F. salimarina* has remained elusive. Herein, we report the genome sequence of strain GTC 22824 isolated from coastal seawater in Osaka, Japan (34.6465° N, 135.3940° E). Approximately 500 mL of seawater was concentrated 50-fold by centrifugation (1,200 × *g* for 20 min) to 10 mL. A 0.2 mL aliquot was inoculated on buffered charcoal yeast extract (BCYE) agar (Kyokuto Pharmaceutical Industrial, Japan) and cultured aerobically at 37°C for 48 h. Small, circular, smooth, grayish-white colonies were obtained and purified by two rounds of subculture on BCYE agar under the same conditions.

The strain was cultured on BCYE agar, and approximately 10–20 colonies were collected, suspended in Tris-EDTA buffer, and pelleted via centrifugation (10,000 × *g* for 5 min). Genomic DNA was extracted using a NucleoBond HMW DNA Kit (Macherey-Nagel, Japan) per the manufacturer’s instructions without any modification.

Long-read sequencing was performed using PromethION 24 (Oxford Nanopore Technologies [ONT], Oxford, UK) and short-read sequencing using NextSeq 1000 (Illumina, San Diego, CA, USA) (3–5). The same DNA extract was used for both. For long-read sequencing, libraries were prepared using a Native Barcoding Kit (SQK-LSK114-24, ONT) without shearing, and small fragments were removed using a Short Read Eliminator XS Kit (Pacific Biosciences, USA). Sequencing was conducted on a FLO-PRO114M flow cell. Base calling used Dorado v7.11.2 (super-accurate mode), and reads were trimmed and quality-filtered using NanoFilt v2.7.1 (6) with “-l 1000 -q 10 –headcrop 50.” For short-read sequencing, libraries were prepared using a DNA Prep (M) Tagmentation Kit (Illumina), followed by 2 × 151 bp paired-end sequencing. Reads were processed with fastp v0.20.1 (7) with “-q 30 -n 20 -t 1 -T 1.” Short-read quality was assessed using fastp (7), and long-read quality was assessed using NanoPlot v1.32.1 (6). Short reads (>95.1% bases > Q30) and long reads (mean quality 22.9) were assembled using Unicycler v0.4.8 (8) with default settings. In this pipeline, terminal overlap trimming and start-position rotation were performed automatically. Genome circularization was confirmed using Bandage v0.8.1 (9) by identifying a single closed-loop topology. Taxonomic integrity was confirmed using Blobtools v1.0 and short-read coverage data (10). Genome annotation was performed using DDBJ Fast Annotation and Submission Tool (DFAST) (11). The assembly was rotated to start with *dnaA* on the forward strand.

Genomic futures are summarized in Table 1. Unicycler and Bandage analyzed and produced a complete circular genome. Genome quality and statistics were assessed using CheckM (v1.2.2) (12) and SeqKit (v2.9.0) (13). CheckM showed 99.57% completeness and 0.62% contamination. DFAST (11) predicted 1,891 coding sequences, including 10 rRNAs and 39 tRNAs, with no CRISPR sequences. Default parameters were used unless otherwise specified. Species identification using average nucleotide identity (ANI) calculated with GTDB-Tk v2.3.2 (14) identified the strain as *Francisella salimarina* (ANI >95%).

**TABLE 1:** Information on the complete genome sequence of the *Francisella salimarina* GTC 22824 strain isolated from coastal seawater in Japan

Parameter	Strain name
	*Francisella salimarina* GTC 22824
NextSeq sequencing^*a*^	
No. of reads	8,214,074
Size (kb)	2,942,155
Avg coverage (×)	617.8
DRA accession no.	DRR909889
ONT sequencing^*a*^	
No. of reads	575,479
Size (kb)	1,746,692.4
Average read length (bp)	3,035
Average coverage (×)	877.2
N50	5,365
DRA accession no.	DRR909890
Assembly	
Assembly N50 (bp)^*c*^	1,991,123
Estimated genome completeness (%)^*d*^	99.57
Estimated genome contamination (%)^*d*^	0.62
Genome structure	One chromosome
DDBJ/GenBank accession no.	AP046135
Genome size (bp)	1,991,123
GC content (%)	
Chromosome	33.0
No. of coding sequences^*b*^	1,891
No. of rRNAs^*b*^	10
No. of tRNAs^*b*^	39
No. of CRISPRs^*b*^	0

^*a*^ DRA, DDBJ Sequence Read Archive.

^*b*^ DFAST, DDBJ Fast Annotation and Submission Tool.

^*c*^ Determined with seqkit v2.9.0.

^*d*^ Determined with CheckM v1.2.2.

## Data Availability

The genome sequence of *F. salimarina* GTC22824 has been deposited in DDBJ under accession no. AP046135. Raw reads have been deposited in the DDBJ Sequence Read Archive under accession nos. DRR909889 and DRR909890.
